# Neonatal pain perception, management and review of practises among medical workers in Nigeria newborn units

**DOI:** 10.4314/ahs.v23i1.72

**Published:** 2023-03

**Authors:** Okonkwo Ikechukwu, Okolo Angela, Omoigberale Augustine

**Affiliations:** 1 Neonatal Division, Department of Child Health, University of Benin Teaching Hospital, Benin City, Edo State, Nigeria; 2 Department of Paediatrics, Federal Medical Centre Asaba, Delta State, Nigeria

**Keywords:** Neonatal pain management, Nigerian newborns, health care workers

## Abstract

**Background:**

Pain is an unpleasant sensory and emotional experience associated with actual or potential tissue damage. The perception of pain is variable and knowledge may not match practise.

**Aims:**

To ascertain the knowledge, attitude and practice of neonatal pain management (NPM) among HCW in newborn units across Nigeria.

**Methods:**

The validated questionnaire administered to consenting doctors and nurses working in various newborn units in Nigeria was utilised.

**Results:**

There were 256 respondents from tertiary institutions 228 (89.1%) located in 31 states of Nigeria. There were 91% doctors and 8.6% nurses'. The perception of newborn pain was high (≥95%) among doctors and nurses . Up to 67.1% of the doctors and 57.1% of nurses were aware of NPM. One third (37.3%) of doctor respondents knew of NPM from friends and colleagues while half of the nurses knew from course textbooks.

Pain definition was in most by sensation (94%), 50% by emotion. Most nurses were aware of the subjective report of potential tissue damage and knew the facial expressions of pain compared to doctors. Crying was equally recognised as an expression of pain. Perception of non-pharmacologic methods of NPM was highest for massaging and KMC; the knowledge of Sucrose analgesia was low. Procedural pain perception was poor and analgesia was for few procedures.

**Conclusions:**

Perception of pain was high but did not match knowledge and practice of NPM. Formal education on NPM was lacking in the training of HCW.

## Introduction

Pain is an unpleasant sensory and emotional experience associated with actual or potential tissue damage.^1^ It is also further defined as a subjective experience that is best understood through self-reports. Verbal communication and self-report are considered the “gold standard” for pain assessment.^2^ Pain is difficult to assess and more challenging in very young or preverbal infants. This is true especially for newborn infants, either term or preterm babies.^3^

Newborn pain is assessed by the physician, nurse or reported by the parents. Pain perception and practice is affected by belief systems, attitudes and parental pain history. They also influence knowledge of pain, impact and coping strategies.^4^–^5^

Pain perception among babies, has been the subject of debate within the medical profession and the perception and intensity of pain in babies has been tested in surveys of newborn healthcare workers with different outcomes. A survey by Porter et al^6^ of health care workers from level II and III newborn nurseries in St. Louis area, USA showed that 59% of physicians and 64% of nurses believe that infants can feel the same amount of pain as adults. Twenty seven percent believed that infants could feel more pain, 10% believed they feel less pain than adults. This varied perception of newborn pain by healthcare workers maybe the cause of diverse acceptance of pain relief.

Pain may arise from normal activities, ill health & diseases or interventions. Health care workers attending to babies and parents' alike ought to recognise pain in babies. Procedural pain is another source of pain in babies; perception and practice are also varied and diverse. Babies admitted into newborn units are more likely to undergo painful therapeutic or diagnostic procedures.^7^–^10^ They include venipuncture for bloodwork or intravenous access, heel lance, umbilical venous cannulation, urinary bladder catheterization, endotracheal intubation or extubation, nasogastric tube insertion, lumbar puncture, circumcision and several other procedures. The perception of pain in babies by their care givers is key to prevent, alleviate or avoid such events. Stevens et al^10^ found that infants born between 27 to 31 weeks gestation received up to 134 painful procedures within the first two weeks of life and approximately 10% of the youngest and/or sickest infants received over 300 painful procedures.^7^ Porter and colleagues^11^ in 1999 found that preterm infants experienced, on average, over 700 painful procedures during their hospitalization. These findings underscore the need to prevent and provide relief from pain and discomfort during these procedures.

Gibbins et al^12^ reported the mean number of painful procedures per day was greater than 5 (range 0 to 10) and 12/day if non-tissue damaging procedures were included. Stevens et al 2005^13^ reported more than 10 as the mean number of painful procedures per day.

Babies feel pain, as every other living being.^6^–^9^ They however cannot verbalize their pain hence they depend on us to recognize, manage and assess their pain. Premature infants <32 weeks may in fact experience higher levels of pain than older infants, even older preterm infants experience more pain than adults. Neonates are more susceptible to the long-term effects of pain.^7^

Although procedural pain management is the responsibility of the physicians and/or nurses who care for hospitalized infants, little is known about their beliefs and behaviour regarding pain management, particularly with regard to procedural pain. The purpose of this study was to examine physicians' and nurses' beliefs regarding infant pain and the perception of the procedures commonly performed, and to examine their perception of what should be done to reduce infant procedural pain.

There are no reports of neonatal pain perception and management from researchers in sub-Saharan Africa. Even though pain has been termed the fifth vital sign in high income countries, the same cannot be said of low resource countries where underdeveloped and weak health systems abound. Pain is a universal challenge, although perception and practise remain diverse.

This study assessed the knowledge, attitude and practice of neonatal pain & its management in newborn nurseries of training institutions and tertiary hospitals in Nigeria.

## Methods

The study was conducted at the University of Benin Teaching Hospital, Benin City, Nigeria during the update course for paediatric resident doctors working in newborn and paediatric units around the country. The nurse respondents were paediatric nurses in training or working in the newborn unit of the University of Benin Teaching hospital and trainee paediatric nurses on rotation in the unit.

A pretested and validated questionnaire with scored and open-ended questions on knowledge, attitude and practice of neonatal pain was administered to consenting medical doctors and nurses. Enquiries about the management of neonatal pain in their respective neonatal units were included.

The responses were weighted according to the status of the respondents and completeness of solicited information. Consultant with neonatal training working in newborn units were considered as key informants. The data gathered were entered into an IBM/ SPSS 20 spreadsheet and analysed.

## Results

Out of 285 questionnaires administered, 256 were returned; a response rate of 89.8%. The 256 respondents were predominantly (89.1%) from tertiary health institutions located in Abuja and 30 states of Nigeria. Benue, Jigawa, Kebbi, Taraba, Yobe, Zamfara states had no respondents. The highest number of respondents 33 (12.9%) were from Ebonyi State, 160 (62.5%) of all the respondents practised in their state capitals. There were 233 (91%) doctors of various cadre and 22 (8.6%) nurse respondents. The distribution and general characteristics of the respondents are shown in Table I.

**Table I:** General characteristics and distribution of respondents

Category of medical worker	n (%)
Doctors	233 (91)
Nurses	22 (8.6)
Unknown	1 (0.4)
**Gender**	
Male	102 (39.8)
Female	150 (58.6)
Unknown	4 (1.6)
**Cadre of doctor respondents**	
Consultants	6 (2.6)
Senior registrars	31 (13.3)
Junior registrars	175 (75.1)
Medical officers	21 (9.0)
**Designation of nurse respondents**	
Chief Nursing Officer	4 (18.2)
Assistant Chief Nursing Officer	1 (4.5)
Senior nursing officer	2 (9.1)
Nursing Officer I	2 (9.1)
Nursing Officer II	10 (45.5)
Pupil Midwife	3 (13.6)
**Type of practise**	
Private	3 (1.2)
Public	239 (93.3)
Unknown	14 (4.5)
**Level of public practice**	
Primary	4 (1.6)
Secondary	7 (2.7)
Tertiary	228 (89.1)

The perception of newborn pain was high among doctors 229/233 (98.3%) and nurses 21/23 (95%). Up to 151/225(67.1%) of doctors and 12/21 (57.1%) nurses’ respondent were aware of neonatal pain management (NPM). About 37.3% of doctor respondents learnt NPM from friends and colleagues and another 43/158 (27.2%) learnt from seminars and academic presentations. One half (50%) of the nurses got information on NPM through textbooks and 16.7% had lectures on NPM.

**Figure 1:**
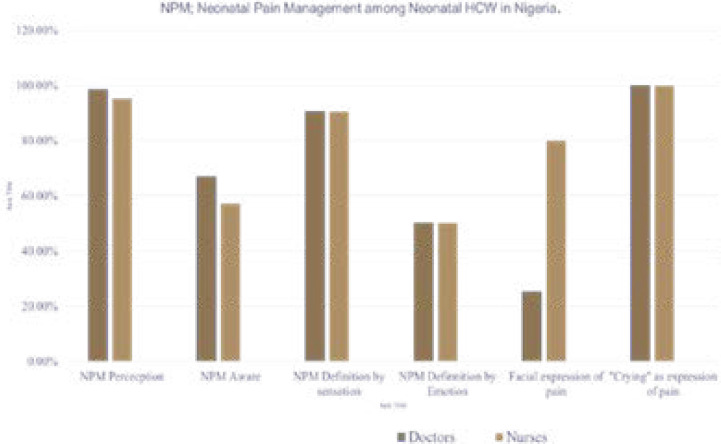
Neonatal pain perception among doctors and nurses.

Neonatal pain was correctly defined using sensation by 90.4% of doctors and nurses, 50% by emotions. About 83.3% of nurses knew pain by the subjective report of potential tissue damage. Eighty percent of nurses knew the facial expressions or coding of pain compared to 25% doctors. Doctors and nurses alike recognised ‘crying’ as expression of pain. This is shown in Figure 1.

**Figure 2:**
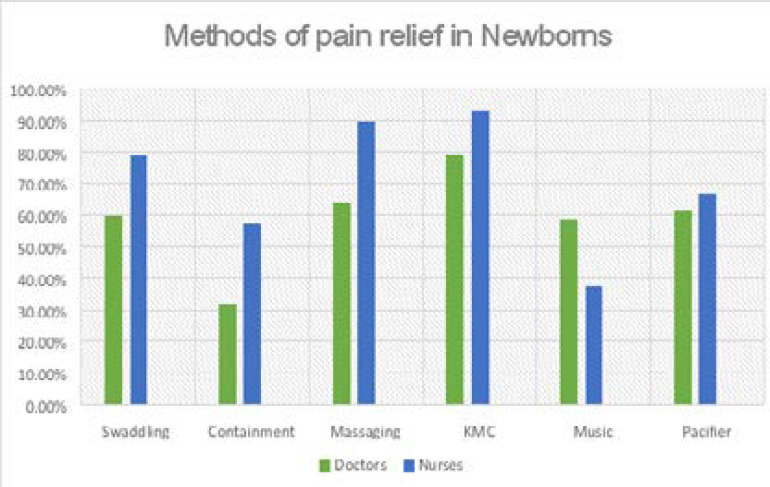
Use of non-pharmacologic methods of pain relief among doctors and nurses.

The knowledge of non-pharmacologic or physical means of pain relief among doctors showed swaddling 59.8%, containment 32.6% and massaging 64.1%. Among the nurses, swaddling was 73.8%, containment 57.1%, massaging 90% and use of KMC 92.9%. This is shown in Figure 2.

Knowledge of the use of pharmacologic means of pain relief such as morphine was known to 48% of doctors, 50% of nurse respondents. Fentanyl was known to 34.1% of doctors and 20% of nurses. Sucrose analgesia for pain relief was known to 27.7% of doctors & only 5.3% of nurse respondents.

On the perception of the types of procedure requiring pain management among doctor respondents; heel lance 32.2%, suctioning 16.7%, and venepuncture 16.7%. Doctors favoured NPM (analgesia) for the following procedures; umbilical catheterization 93.3%, chest tube insertion 82.4%, lumber puncture and circumcision 93.3%.

Forty two percent of doctors and 47.1% nurses' agreed that pain could be quantified. Knowledge of the premature infant's pain profile was 64.6% among doctors and 66.7% of nurses did not know about the scale.

Up to 60.4% of doctors did not know the facial coding Scale. The neonatal cry score was not known to 54.6% of doctor and 50% of nurse respondents.

Doctors (93.3%) and nurses (100%) respondent agreed that NPM is important and should be implemented in newborn units in Nigeria.

## Discussion

Neonatal pain management is yet to be considered a topical issue in most sub-Saharan countries compared to developed countries^14^ as evidenced by absence or paucity of published protocols or guidelines from the sub-region. This maybe reinforced by absence of institutional protocols or guidelines for identification, prevention and management of neonatal pain among neonatal units from which the respondents were surveyed. Neonatal pain therefore is yet to be discussed commonly among paediatricians in Nigeria. This may be due to the overwhelming burden of infections, prematurity and asphyxia which account for more than three quarters of the national newborn mortality^15^ Emphasis on improvement in the direct causes of newborn survival might be responsible for the neglect of quality-of-life issues like neonatal pain management. The poor perception, knowledge and practice of neonatal pain is also a reflection of the cultural, religious, social and religious values in many resources limited settings.^4^–^5^

Pain perception in this survey was high among doctors and nurses, however perception did not match with the practice of NPM. This may be partly due to absence of training on NPM as part of undergraduate and postgraduate training curriculum of medical and nursing personnel. Furthermore, attitude to pain relief is discretionary even when it is recognised as an unpleasant experience. This survey highlights the need to raise awareness, level of knowledge and practice of pain relief in our locale. The doctor and nurse respondents in this survey showed a high concordance in NPM perception, definition by sensation, emotion and crying as an expression of pain, nurse respondents fared better at the recognition of facial expression of pain in babies. The survey clearly shows the heightened awareness and preference for non-pharmacologic means of pain relief than the pharmacologic methods. A higher proportion of nurse respondents had better awareness of non-pharmacologic methods of pain relief and the converse was true among doctors for pharmacologic methods. The knowledge and use of pharmacologic methods of pain relief among doctors and nurses were low for morphine and fentanyl and very low for sucrose analgesia.

The perception of procedural pain relief was poor for heel lance, venipuncture and suctioning but pain perception was good for circumcision, lumbar puncture and umbilical catheterization and chest tube insertion. These findings may be an indication of the health system status regarding neonatal pain management. The paucity of similar studies or surveys from the sub region make comparison with this study's findings even more challenging. That babies do feel pain is not in doubt but perception is poor about the several causes of neonatal pain especially among those admitted to the neonatal unit. The practice of NPM is even more challenging with the absence of commonly used pharmacologic agents like Eutectic Mixture of Local Anaesthetics (EMLA) and oral sucrose solution from the surveyed centres as is the norm in neonatal units of high-income countries. However, some non-pharmacologic methods like breastfeeding, kangaroo mother care (KMC), non-nutritive sucking and swaddling are available and useful for procedural pain relief in resource limited settings. These have been shown to reduce acute procedural pain compared to no treatment group.^16^ The highest perception (>90%) for use of non-pharmacologic method of pain relief was massaging and KMC by nurse respondents. This finding maybe related to the traditional acceptance and practice of newborn massaging and sustained skin to skin contact by carrying babies on their backs (backing) in the study locale by women. KMC or Skin-to-skin care (SSC) diminishes pain responses in term and preterm neonates and supports the recovery following completion of painful procedures.^17^ Out of six non-pharmacologic methods of NPM, music therapy which is a newer method was the method noted with higher perception by doctors than nurse's respondent. There is insufficient evidence to support music as a pain reduction strategy in newborns;^18^,^19^; however, it may be used to support developmental care.

Newborn babies both term and preterm, experience pain and should receive safe, efficient and effective pain relief.^20^ For pain to be more effectively managed, there is need for pain to be considered the fifth vital sign. The strategies for neonatal pain management needs to be proactive and begin with the right perception, knowledge and practice. When compared with adults, the newborn displays hypersensitivity to sensory stimuli and as such, is more prone to pain and its consequences.^6^ Newborns cannot verbalize their pain and thus depend on others to recognize, assess and manage their pain, hence the role of the caregiver and healthcare worker.

The strength of this study includes the high response rate (89.8%) compared to 71% and 80% in similar studies assessing neonatal pain management.^6^,^21^ The reason for the seemingly high return rate is not apparent to the researchers.

All the respondents were unanimous in their perception that newborn babies feel pain, similar finding was reported by da Lima et al 1996^21^ among paediatric anaesthetists in United Kingdom and Ireland. This finding was a change in perception and practice from the original survey done 7years previously.^22^ It was noted that in contrast to the present survey, 13% of the respondents thought that newborn infants and 7% that neonates did not feel pain. This may be a surprise finding among anaesthetists whose training is on analgesia and anesthesia. That was a landmark report of change in pain perception as before 1986, neonates and infants were assumed to be incapable of perceiving pain and seldom given analgesics for operations.^22^,^23^The long-held medical myth that neonates were unable to experience pain was clearly dispelled by the work of Anand and Hickey, who described the potential mechanisms of pain in babies.^24^

There are regularly updated guidelines and detailed protocols local, national and regional for the assessment, prevention and management of neonatal pain in high-income countries.^25^,^26^

The perception of neonatal HCW to procedural pain was also assessed by this study of six commonly performed procedures in contrast to 12 accessed by Porter et al.^6^ The perception determines administration of analgesia or anaesthesia as needed. The perception of pain among doctors for venipuncture and suctioning was very poor (<20%) while it was poor (32%) for heel lance in this study. These are procedures where non-pharmacologic measures can easily be used to achieve comfort during these procedures in settings where pain perception is high.^27^ The high preference for pharmacologic analgesia by respondents (>82-94%) for umbilical catheterization, chestube insertion, lumber puncture and circumcision is of note. This perception was similarly noted by Porter et al6 and da Lima et al^21^ where these procedures were classified as minor surgeries.

Adequate pain management begins with effective pain assessment.^28^ The pain assessment scale allows the clinician to quickly assess and successfully manage the pain experiences of both preterm and term babies. Awareness of pain assessment in this survey was poor (<50%); there was also moderate awareness of pain assessment tools among doctors and poor awareness among nurses. This maybe one of the barriers to routine newborn pain assessment in our locale after more than quarter of a decade after development of systematic pain assessment tools for neonates. More than 40 scales have been developed to evaluate pain in this fragile, non-verbal population.^29^,^30^ Doctor respondents seem to have better knowledge of newborn pain assessment tools than nurses in this survey. More nurses however agreed than doctors that NPM is important and should be implemented in their newborn units.

NPM should be proactive and not reactive hence the need to form neonatal pain interest and advocacy groups to enhance the study, dissemination and implementation of NPM in the study locale. There is an urgent need for newborn Pai assessment and management guidelines in sub-Saharan African countries like Nigeria for routine care and procedural pain management. These efforts may be championed by paediatric, neonatal, anaesthesia or analgesia professional associations with cooperation of interest and advocacy groups or development partners. In conclusion, babies feel pain, but the perception of neonatal pain is poor and intervention sparse among neonatal health care workers in Nigeria. The perception of pain among HCW was variable and knowledge does not match practise. Formal education on NPM was lacking in the training of the healthcare personnel. Attitude to relief of newborn pain was discretionary despite the knowledge of its definitions. There is need to raise the level of knowledge and practice for NPM in our low resource setting by providing local guidelines and modular trainings for healthcare workers.
